# Elevated levels of CXCL10 in the Periodic Fever, Aphthous stomatitis, Pharyngitis and cervical Adenitis syndrome (PFAPA) during and between febrile episodes; an indication of a persistent activation of the innate immune system

**DOI:** 10.1186/1546-0096-11-38

**Published:** 2013-10-17

**Authors:** Jostein Førsvoll, Einar Klæboe Kristoffersen, Knut Øymar

**Affiliations:** 1 Department of Pediatrics, Stavanger University Hospital, Postboks 8100, 4068, Stavanger, Norway; 2 Department of Clinical Science, University of Bergen, Bergen, Norway; 3 Department of Immunology and Transfusion Medicine, Haukeland University Hospital, Bergen, Norway

**Keywords:** PFAPA, Cytokines, Periodic fever, IL-6, CXCL10, CCL4, IP-10, MIP-1β, Lymphocytes

## Abstract

**Background:**

The Periodic Fever, Aphthous stomatitis, Pharyngitis and cervical Adenitis syndrome (PFAPA) is the most common periodic fever syndrome in childhood. Clinically, PFAPA may resemble autoinflammatory diseases, but the etiology is not fully understood.

**Methods:**

We measured inflammatory proteins in plasma and hematologic parameters in children with PFAPA during and between febrile episodes, and in a control group with suspected bacterial pneumonia. In children with PFAPA, a first blood sample was taken within 24 hours of a febrile episode and a second sample between episodes. In children with pneumonia, the first sample was taken shortly after admission and a second sample after full recovery.

**Results:**

A total of 22 children with PFAPA and 14 children with pneumonia were included. In children with PFAPA, levels of interleukin (IL) 6, CXCL10 and CCL4 were significantly increased during febrile episodes. The levels of IL-6 and CXCL10 were higher in children with PFAPA during febrile episodes than in children with pneumonia. The levels of CXCL10 remained higher in children with PFAPA between febrile episodes compared to children with pneumonia after recovery. Children with PFAPA had a relative eosinopenia and lymphocytopenia with reduced numbers of both CD4+ and CD8+ T cells during febrile episodes. This pattern was not observed in the children with pneumonia.

**Conclusions:**

The results indicate an innate immune response as the initial step in PFAPA, and a subsequent adaptive response with activation and redistribution of T cells. Moreover, an activation of the innate immune system involving CXCL10 may persist between febrile episodes. CXCL10 may be a possibly clinical marker in children with PFAPA.

## Background

The syndrome of Periodic Fever, Aphthous stomatitis, Pharyngitis and cervical. Adenitis (PFAPA) was first described as a clinical entity by Marshall et al. in 1987 [1]. PFAPA is an inflammatory disorder mainly of early childhood, with periodic fever accompanied by a predictable set of symptoms with aphthous stomatitis, pharyngitis and cervical adenitis being the most frequent. The most commonly used clinical diagnostic criteria were suggested by Thomas et al. in 1999 [2]. PFAPA has been reported from different ethnic groups [3], and recently we reported a yearly incidence of 2.3 per 10.000 children under the age of five in Norwegian children [4].

The lack of a microbiological agent as the cause of fever [1,2], a well-documented abrupt response to a single dose of cortisone [5,6] and no evidence of autoimmunity have led to the hypothesis that PFAPA is an autoinflammatory disease [7-10]. For some of the autoinflammatory diseases the genetic and pathophysiological mechanisms have been revealed, but for PFAPA and others this remains unclear [7].

The pathophysiology of PFAPA has been explored in four studies through different approaches [8-11]. In all four studies cytokines were measured in serum, but with different panels and different time-relation to the fever episodes. Except for a clear increase of serum interleukin (IL) -6 during the febrile phase of PFAPA, no uniform results have been found. In two studies however, an increase of the chemokines CXCL9 and CXCL10 during the febrile phase was also reported [8,9]. In two studies IL-1β has been suggested to play a pivotal role in PFAPA [9,10].

During the febrile phase of PFAPA, the immunological response may mimic that of bacterial infections with high levels of C-reactive protein (CRP) [12,13]. With the exception of one study which included children with hereditary periodic fever syndromes during febrile attacks as controls [9], only control groups of healthy children were included in the studies of the etiology of PFAPA [8,10,11]. This may limit the ability to study the PFAPA-specific immunological characteristics during inflammation.

Our aim was to study the immunological and haematological characteristics in PFAPA during febrile episodes and recovery, and include a control group with a well-defined bacterial infection.

## Methods

### Study population

Since 2004, children with PFAPA diagnosed at Stavanger University Hospital have been included in a prospective study [4], and children included between 1st of January 2008 and 31st of August 2011 were invited to participate in the present immunological study.

The diagnosis of PFAPA was set according to the criteria suggested by Thomas et al. [2] as described in detail elsewhere [4]. The children should not be suspected for or diagnosed with any other autoinflammatory or other disease as the reason for recurrent fever. Cyclic neutropenia was excluded in all patients by serial measurements of neutrophils two times weekly between two subsequent attacks. During the febrile attacks the children were evaluated by one of the authors (JF or KØ). A throat culture was collected from all children during attacks; these were negative in all children except for one child who was excluded from the study. Children admitted to Stavanger University Hospital with clinical symptoms of pneumonia, radiological verified consolidation and CRP ≥ 150 mg/L and with no chronic condition making theme prone to infections were recruited as controls.

The study was approved by the regional committee of research ethics, and an informed consent was obtained from parents of all children.

### Laboratory analyses

In children with PFAPA, a blood sample was taken within 24 hours of the onset of a typical febrile episode (PFAPA-f) and another (non-febrile) blood sample was taken subsequently between two febrile episodes (PFAPA-nf), after at least 10 days without fever. If only one of two samples was taken, the child was also included.

In children with pneumonia (Pn), blood samples were taken as soon as possible after the diagnosis was set after admission (Pn-f). In most patients, a second blood sample was taken after complete resolution of their infection, at least four weeks after full recovery (Pn-nf).

Systemic steroids were not given to children with PFAPA or pneumonia during the study period. Non-steroid anti-inflammatory drugs were only administrated after the blood samples had been taken, except for one child with pneumonia who had received one dose of ibuprofen 34 hours prior to admission.

White blood cell count with subgroups of neutrophils, monocytes, lymphocytes and eosinophils and thrombocytes were analysed on a Sysmex XE-5000 (Kobe, Japan) as a part of routine work-up.

Lymphocyte subpopulation quantifications were performed using the BD Multitest 6-color TBNK kit with BD Trucount Tubes for relative and absolute concentration determination (BD Biosciences, San Jose, CA, USA). The samples were prepared according to the manufacturer’s instructions and analyzed on a BD Canto II flow cytometer (BD Biosciences) using BD Canto 2.1 analysis software.

In order to avoid ex vivo release or production of cytokines after sampling, a citrate-coated collection tube was immediately cooled and centrifuged by the use of a Kubota 5930 centrifuge at 3300 rpm for eight minutes at 4°C. After centrifugation, the citrate plasma was pipetted off and frozen at -80°C. Samples were thawed and divided when analysed for cytokines. The part of the samples not used for cytokine analysis was refrozen and stored.

Cytokines in serum were analysed using a 27-plex cytokine panel (BioRad, CA, USA) and a Luminex-based reader (Luminex Corporation, Texas, USA). Samples and standards were prepared and analysed according to the manufacturer’s instructions. The analyses were performed in duplex. Manufacturer derived detection limit ranged from 0.2-14.6 pg/ml depending on the cytokine (See Table 1 for list of cytokines with the corresponding level of detection). When available, the IUPHAR-nomenclature was used for cytokines and chemokines [14].

**Table 1:** Levels of cytokines in children with the periodic fever, aphthous stomatitis, pharyngitis and cervical adenitis syndrome (PFAPA) and children with pneumonia (Pn)

	**Pn-nf**	**p-value**	**PFAPA-nf**	**p-value**	**PFAPA-f**	**p-value**	**Pn-f**
		**Pn-nf**		**PFAPA-f**		**PFAPA-f**	
		**vs**		**vs**		**vs**	
		**PFAPA-nf**		**PFAPA-nf**		**Pn-f**	
	**(n=10)**		**(n=21)**	**(n=13)**	**(n=14)**		**(n=14)**
IL-1β	nd	0.004	2.5	ns	2.6	0.002	nd
(LoD: 0.8)			(0.1, 5.4)		(0.9, 5.1)		
CXCL10	731.9	0.000	1762.5	.003	4787.2	0.002	1512.7
(LoD: 6.5)	(582.7, 1038.0)		(1305.6, 3401.0)		(2801.1, 7743.5)		(685.5, 3179.4)
IL-6	15.4	ns	11.2	.001	49.6	0.035	21.7
(LoD: 1.1)	(9.5, 23.7		(4.8, 19.0)		(21.1, 103.8)		(18.3, 29.7)
CCL4	70.5	ns	52.8	.019	63.0	ns	59.4
(LoD: 1.1)	(52.9, 83.6)		(30.3, 77.2)		(47.5, 93.4)		(44.7, 75.0)
IL-17	41.6	ns	17.4	ns	7.4	0.002	64.5
(LoD: 0.2)	(25.4, 135.7)		(0.7, 54.6)		(0.7, 29.5)		(26.6, 81.2)
CCL5	6878.3	0.017	3197.5	ns	3007.8	0.016	4856.7
(LoD: 1.2)	(4066.2, 7154.3)		(2200.8, 4694.1)		(1864.5, 4019.3)		(3028.0, 6916.8)
IL-1ra	234.0	ns	129.3	ns	121.5	0.002	249.3
(LoD: 1.4)	(116.4, 368.1)		(69.5, 196.8)		(95.7, 170.8)		(176.7, 328.9)
IL-2	17.6	0.010	nd	ns	nd	0.000	17.6
(LoD: 1.1)	(7.3, 29.9)						(9.2, 21.7)
IL-9	24.4	ns	4.0	ns	6.0	0.011	29.7
(LoD: 0.7)	(11.6, 77.5)		(0.6, 30.8)		(0.5, 17.7)		(15.3, 41.3)
CCL11	65.0	ns	20.8	ns	nd	0.007	59.3
(LoD: 14.6)	(10.4, 133.1)		(0.3, 71.6)				(14.7, 115.1)
FGF-2	35.9	ns	24.0	ns	26.4	0.044	44.7
(LoD: 6.8)	(24.5, 63.2)		(4.8, 47.7)		(14.1, 40.6)		(27.4, 55.7)
G-CSF	74.0	ns	92.0	ns	126.9	0.016	94.0
(LoD: 1.1)	(54.8, 103.4)		(61.6, 111.9)		(95.9, 169.0)		(55.6, 106.9)
CCL2	38.0	ns	36.1	ns	38.0	0.039	27.6
(LoD: 6.8)	(34.5, 41.9)		(26.2, 42.1)		(28.4, 54.5)		(19.2, 32.9)
CCL3	15.5	ns	3.0	ns	4.2	0.004	16.9
(LoD: 2.4)	(8.2, 21.5)		(0.2, 14.8)		(0.4, 7.0)		(13.6, 18.9)
PDGF-BB	1440.5	0.025	532.4	ns	406.0	0.004	1284.0
(LoD: 1.0)	(529.7, 1942.0)		(301.5, 910.1)		(263.2, 1093.6)		(1056.0, 2813.3)
CXCL8 (IL-8)	19.1	ns	20.1	ns	19.0	ns	24.3
(LoD: 0.5)	(12.7, 32.3)		(14.0, 29.9)		(15.6, 23.4)		(18.5, 29.1)
INF-γ	198.9	ns	279.4	ns	323.5	ns	226.6
(LoD: 19.3)	(125.3, 336.9)		(208.3, 418.9)		(235.3, 437.4)		(166.7, 306.3)
IL-4	5.7	ns	7.0	ns	6.8	ns	6.8
(LoD: 0.5)	(3.1, 8.2)		(4.9, 9.8)		(4.9, 9.4)		(4.4, 8.2)
IL-5	6.3	ns	5.7	ns	5.8	ns	12.0
(LoD: 0.8)	(1.8, 21.2)		(3.1, 9.4)		(4.5, 9.3)		(1.8, 18.3)
IL-7	19.9	ns	25.9	ns	25.4	ns	19.5
(LoD: 0.5)	(11.5, 26.3)		(19.1, 31.1)		(19.4, 29.6)		(12.5, 26.3)
IL-10	13.1	ns	6.5	ns	8.1	ns	8.3
(LoD: 0.9)	(1.6, 24.9)		(3.2, 13.7)		(4.3, 11.1)		(1.2, 16.8)
IL-12p70	46.1	ns	25.8	ns	24.5	ns	40.4
(LoD: 0.5)	(18.5, 89.5)		(12.2, 40.8)		(18.1, 33.4)		(14.2, 72.2)
IL-13	16.9	ns	10.1	ns	10.4	ns	13.3
(LoD: 2.1)	(8.3, 36.1)		(6.4, 17.5)		(7.6, 14.0)		(7.2, 21.9)
IL-15	nd	ns	nd	ns	nd	ns	nd
(LoD: 4.2)							
GM-CSF	nd	ns	nd	ns	nd	ns	nd
(LoD: 4.5)							
TNF-α	66.2	ns	67.1	ns	68.1	ns	82.5
(LoD: 3.0)	(40.6, 126.8)		(42.9, 101.6)		(56.3, 113.1)		(55.9, 116.2)
VEGF	43.2	ns	22.3	ns	30.6	ns	38.3
(LoD: 0.5)	(24.4, 70.0)		(9.2, 47.1)		(9.1, 43.4)		(21.7, 87.3)

Values are given as pg/ml and presented as median (quartiles).

Pn-nf: Children with pneumonia after complete remission.

PFAPA-nf: Children with PFAPA between febrile episodes.

PFAPA-f: Children with PFAPA during febrile episodes.

Pn-f: Children with pneumonia shortly after admission.

LoD: Limit of detection as stated by the manufacturer.

ns: not significant.

nd: not detectable.

Due to observed fluctuations in the subsets of leucocytes and cytokine pattern, samples were later analysed for soluble IL-2 receptor alpha (sCD25) and sCD163 collectively.

sCD25 was analysed using a Quantikine Elisa Human Soluble IL-2 receptor alpha Immunoassay (R&D Systems, Minneapolis, USA). Samples and standards were prepared and analysed according to the manufacturer’s instructions. The minimal detectable dose is typically less than 10 pg/ml according to the manufacturer.

sCD163 was analysed using a Quantikine Elisa Human CD163 Immunoassay (R&D Systems). Samples and standards were prepared and analysed according to the manufacturer’s instructions. Sensitivity as stated by the manufacturer: Forty-six assays were evaluated and the minimum detectable dose of CD163 ranged from 0.058-0.613 ng/ml. sCD25 and sCD163 samples were analysed singly.

Serum immunoglobulin G (IgG), IgA, and IgM were measured using a Siemens BN ProSpec Nephelometer. Serum IgD was measured by radial immunodiffusion, (IgD RID kit – NL, Binding Site, Birmingham, UK).

### Statistical analyses

Values below standard range were accepted as they were, but if the median value was below the level of detection the results were referred to as not detectable. Values out of range were set as the lowest and highest values detected by the kit respectively.

Comparisons between groups were analysed by the chi-square test and non-parametric Mann–Whitney U test. When comparing samples from febrile and non-febrile children within groups, only children with paired samples were included and the results were analyzed with the Wilcoxon Signed Ranks Test. P-values of <0.05 were regarded as statistically significant. All statistical analyses were performed using the IBM-SPSS version 20 statistical package.

## Results

Twenty-two children diagnosed with PFAPA were included in the study. In 13 of these, blood samples were collected both during (PFAPA-f) an in between (PFAPA-nf) febrile episodes. In eight children, samples were taken only during the non-febrile phase, and in one patient a sample was taken only during the febrile phase.

Fourteen children with pneumonia were included. In 10 of these, samples were taken both during the febrile (Pn-f) phase and after full recovery (Pn-nf). In four children, a sample was taken only during the febrile phase (Pn-f). The characteristics of the children with PFAPA and pneumonia are given in Table 2.

**Table 2:** Clinical characteristics of children with the periodic fever, aphthous stomatitis, pharyngitis and cervical adenitis syndrome (PFAPA) and children with pneumonia

	**PFAPA (n = 22)**	**Pneumonia (n = 14)**	**p-values**
Gender (boys / girls)	14 / 8	7 / 7	0.418
Age at time of sampling (months) †	36.5 (23.8, 62.6)	34.1 (17.5, 51.1)	0.860
Duration of fever at time of sampling (hours)	14.0 (11.5, 16.5)‡	96.0 (60.0, 135)	<0.001
Age of onset (months)	9.5 (4.3, 12.0)		
Duration of febrile episodes (days)	3.8 (3.0, 5.0)		

Results are given as median (quartiles) except for gender.

†Age at first sample in all children.

‡n = 14.

### Leucocyte counts

**Figure 1:**
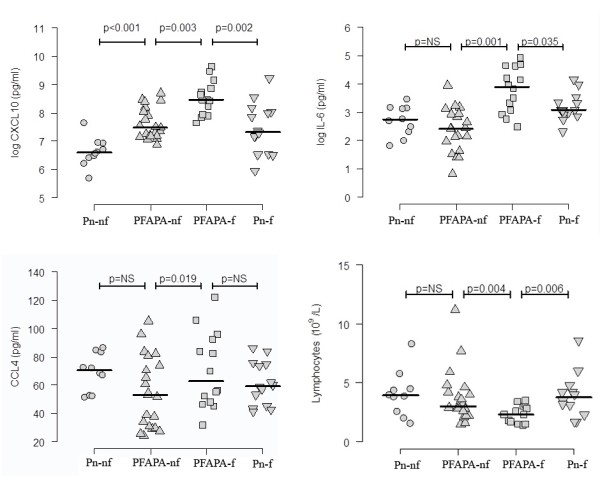
**Levels of CXCL10, Interleukin (IL) 6, CCL4 and number of lymphocytes in children with PFAPA during (f) and outside febrile episodes (nf), and in children with pneumonia (Pn) during the episode (f) and after recovery (nf).** The line represents the median value. CXCL10 and IL-6 are presented with a log scale.

Levels of leucocytes are presented in Table 3, and the distribution of lymphocytes in Figure 1. The white blood cell count, neutrophil count and monocyte count were significantly higher and the lymphocyte and eosinophil count was significantly lower in PFAPA-f compared to PFAPA-nf. No significant differences were observed for thrombocytes. The lymphocyte and eosinophil count in PFAPA-f was significantly lower than in Pn-f, but no significant difference was observed between non-febrile samples (PFAPA-nf and Pn-nf). The lymphocyte and eosinophil count did not differ between Pn-f and Pn-nf.

**Table 3:** Haematological variables and lymphocyte populations in children with the periodic fever, aphthous stomatitis, pharyngitis and cervical adenitis syndrome (PFAPA) and children with pneumonia (Pn)

	**Pn-nf**	**p-value**	**PFAPA-nf**	**p-value**	**PFAPA-f**	**p-value**	**Pn-f**
		**Pn-nf**		**PFAPA-nf**		**PFAPA-f**	
		**vs**		**vs**		**vs**	
		**PFAPA-nf**		**PFAPA-f**		**Pn-f**	
	(n=10)		(n= 21)	(n=13)	(n=14)		(n=14/12*)
White blood cells (10^9^/L)	9.2	ns	7.9	0.003	13.3	ns	14.7
	(6.1, 11.4)		(5.7, 10.1)		(9.4, 15.2)		(10.5, 19.2)
Neutrophils (10^9^/L)	3.15	ns	3.2	0.002	9.2	ns	9.1
	(2.3, 5.6)		(2.5,4.1)		(5.8, 11.8)		(5.0, 14.7)
Thrombocytes (10^9^/L)	366	ns	355	ns	299	ns	385
	(289, 413)		(296, 427)		(269, 353)		(275, 482)
Lymphocytes (10^9^/L)	3.95	ns	3.0	0.004	2.3	0.006	3.8
	(2.5, 4.8)		(2.4, 4.4)		(1.5,3.1)		(2.5, 4.6)
Monocytes (10^9^/L)	0.7	ns	0.6	0.002	1.4	ns	1.3
	(0.5, 0.8)		(0.6, 0.8)		(1.2, 1.8)		(1.1, 1.6)
Eosinophils (10^9^/L)	0.2	ns	0.2	0.002	0.0	0.009	0.2
	(0.1, 0.5)		(0.1, 0.4)		(0.0, 0.1)		(0.03, 0.3)
	(n=9)		(n=15)	(n=8)	(n=8)		(n=10)
CD3 (10^6^/L)	2583	ns	2527	0.012	1087	0.021	2145
	(1744, 3098)		(1998, 3006)		(894, 1592)		(1211, 3368)
CD4 (10^6^/L)	1294	ns	1478	0.017	698	0.041	1310
	(1034, 1619)		(1025, 1909)		(494, 1062)		(765, 2006)
CD8 (10^6^/L)	1196	ns	891	0.012	359	0.010	801
	(655, 1325)		(658, 1022)		(254, 420)		(399, 1084)
CD56 (10^6^/L)	253	ns	414	ns	203	ns	172
	(193, 354)		(224, 590)		(116, 329)		(62.5, 303)
CD19 (10^6^/L)	629	ns	596	ns	574	ns	991
	(403, 880)		(430, 1247)		(346, 823)		(393, 1109)

Results are given as median (quartiles).

Pn-nf: Children with pneumonia after complete remission.

PFAPA-nf: Children with PFAPA between febrile episodes.

PFAPA-f: Children with PFAPA during febrile episodes.

Pn-f: Children with pneumonia shortly after admission.

ns: not significant.

* Lymphocytes, monocytes and eosinophils were measured in 12 children with Pn-f.

### Flow cytometric immunophenotyping of lymphocytes

Subsets of lymphocytes are presented in Table 3. The level of CD4+ and CD8+ T-lymphocytes were significantly lower in PFAPA-f compared to PFAPA-nf, and significantly lower in PFAPA-f compared to Pn-f. The levels of all subsets measured by immunophenotyping were similar in PFAPA-nf and Pn-nf. No differences were observed between any of the groups for CD19+ and CD56+ positive lymphocytes.

### Cytokines

Levels of cytokines in plasma are presented in Table 1 and Figure 1. Levels of CXCL10, CCL4 and IL-6 were higher in PFAPA-f than in PFAPA-nf. Levels of IL-1β were low but detectable in children with PFAPA, but undetectable in children with pneumonia. The levels of IL-1β and the other 23 cytokines did not differ between PFAPA-f and PFAPA-nf.

Levels of IL-1β, CXCL10, IL-6, G-CSF and CCL2 were significantly higher in PFAPA-f compared to Pn-f. CXCL10 was significantly higher in PFAPA-nf compared to Pn-nf. CCL5, IL-2 and PDGF-BB were significantly lower in PFAPA-nf than in Pn-nf.

IL-17, CCL5, IL-1ra, IL-2, IL-9, CCL11, FGF-2, CCL3 and PDGF-BB were significantly lower in PFAPA-f than in Pn-f.

For the other cytokines no significant differences were found between the groups.

### sCD25 and sCD163

The measured levels of sCD25 were significantly higher in PFAPA-f compared to PFAPA-nf.

There were no significant differences between PFAPA-f and Pn-f or between PFAPA-nf and Pn-nf. Soluble CD163 did not differ between any of the groups (Table 4).

**Table 4:** Levels of soluble IL-2 receptor alpha (sCD25) and sCD163 in children with the periodic fever, aphthous stomatitis, pharyngitis and cervical adenitis syndrome (PFAPA) and children with pneumonia (Pn)

	**Pn-nf**	**PFAPA-nf**	**PFAPA-f**	**Pn-f**
	**(n=10)**	**(n=20)**	(n=14/13*)	(n=14/12*)
sCD25 (pg/ml)	1162.3	1883.4	2980.2	2297.6
	(820.4, 2110.3)	(970.4, 2458.5)	(1032.4, 3624.4)	(1799.2, 3381.3)
sCD163 (ng/ml)	568.8	665.8	695.4	505.3
	(408.5, 735.0)	(555.7, 844.4)	(528.0, 813.0)	(391.3, 837.1)

Results are given as median (quartiles).

Pn-nf: Children with pneumonia after complete remission.

PFAPA-nf: Children with PFAPA between febrile episodes.

PFAPA-f: Children with PFAPA during febrile episodes.

Pn-f: Children with pneumonia shortly after admission.

For sCD25 measurements the p-value was 0.013 for PFAPA-f versus PFAPA-nf, and the p-value was 0.022 for Pn-f versus Pn-nf.

No other significant differences were found for sCD25 or sCD163.

* sCD163 was measured in 13 children with PFAPA and in 12 children with pneumonia.

### Immunoglobulins

For children with PFAPA, levels of IgG, IgM, and IgA were within normal levels and did not differ from children with pneumonia, and levels of immunoglobulin D were within the age specific normal range for all children [15]. For children with PFAPA, the median value of IgD was 13.0 mg/L (quartiles: 12.9, 39.2).

## Discussion

In this study we found increased levels of the pro-inflammatory cytokines CXCL10, CCL4 and IL-6 during febrile episodes in children with PFAPA compared to non-febrile periods. These cytokines have in common that they are produced in abundant amounts by cells of the innate immune system. Although the cellular origin of a cytokine measured in peripheral blood is unknown, most cytokines can be induced by more than one cell type, the observed pattern of cytokines in this study substantiate an innate trigger in PFAPA. The levels of CXCL10 were higher in PFAPA even between febrile episodes, suggesting a persistent activation of the immune system.

The lymphocyte and eosinophil count in blood decreased and the numbers of both CD4+ and CD8+ T cells were lower during febrile episodes. The observed fluctuations in T cells indicate an adaptive activation and recruitment of these cells to secondary lymphoid organs. CXCL10 and CCL4 are chemo-attractants and may act as link between an innate and an adaptive immune response in PFAPA.

CXCL10 is a small chemokine (a chemotactic cytokine) formerly denoted *interferon-γ induced protein 10*. CXCL10 is one of three known ligands for the CXCR3 chemokine-receptor [16]. Under the influence of interferon-γ, many different cell-types like monocytes, neutrophils, astrocytes, endothelial cells, keratinocytes and fibroblasts can produce CXCL10, but seemingly CXCL10 can also be induced directly by early innate mechanisms [17,18]. CXCL10 is a powerful chemo-attractant for both CD4+ and CD8+ T cells to the site of inflammation, and is expressed in various Th1 type inflammatory diseases [17], and has also been implicated in autoimmune disease [19].

CCL4 was formerly denoted *macrophage inflammatory protein 1β* and is also a chemokine with pro-inflammatory effects [20]. As CXCL10, CCL4 is a chemo-attractant for T cells. CCL4 is produced as a result of an early innate stimulus by activated macrophages and also by a variety of other activated immune cells [18,20].

IL-6 is an important pro-inflammatory cytokine and has many different cellular targets. Macrophages, dendritic cells and monocytes are the most important source of IL-6, and the abundant IL-6 release observed in PFAPA also indicates trigging of the innate immune system [21]. Like IL-1β, IL-6 induces the production of acute phase proteins, pyrexia and sickness behaviour [21,22]. The increased levels of CRP and serum amoyloid A, high fever and malaise following febrile attacks of PFAPA [4,8,9,12] may be attributed to the increase of IL-6.

The elevation of CXCL10 and IL-6 during febrile episodes of PFAPA has been showed in three previous studies [8-10] and elevated CCL4 in one study [9]. An immune response can be characterised on the basis of the cytokines produced by helper T cells after activation. IL-2 and INF-γ are signature cytokines of a Th1 like immune response [23]. The elevation of CXCL10 in the absence of increased Th2- and Th17 cytokines has been used as an argument for a Th1 like immune response in PFAPA [8-10]. This study reports a similar pattern, but also as in previous studies [8,9] we did not find an increase in Th1 signature cytokines such as INF-γ or IL-2 during the febrile attacks of PFAPA. However, the increase in CXCL10 may suggest a mechanism for activation and recruitment of T-lymphocytes to local tissues, as suggested by the alterations in CD4+ and CD8+ lymphocytes.

It has been a question whether the inflammatory process in PFAPA to some extent persists between flares. Brown found thrombocytosis in the afebrile interval, whereas Stojanov et al. in 2006 reported higher levels of serum IL-1β, IL-6, TNF-α and IL-12p70 in the afebrile interval of PFAPA compared to healthy controls [8,11]. In 2011, Stojanov et al. reported higher levels of CXCL9, CCL4, IL-6 and G-CSF in the afebrile interval of PFAPA compared to controls [9]. We could not confirm these results. However, a novel finding in our study is that levels of CXCL10 were higher in children with PFAPA than in the control group also outside the febrile episode. Interestingly, Stojanov et al. also reported higher levels of CXCL10 and G-CSF during febrile attacks of PFAPA than during febrile attacks of other defined hereditary fever syndromes [9]. Together, these results may indicate that pathways involving CXCL10 play an important role in the etiology of PFAPA, and that this process may be active also between the febrile episodes. This could possibly have a genetic background. Finally, CXCL10 may be further studied as a possibly clinical marker in children with PFAPA.

Differences in cytokine patterns between children with PFAPA and controls have also been found between flares. Stojanov et al. found lower levels of IL-4 at all times in children with PFAPA compared to controls, which may be consistent with the recent finding that IL-4 cytokine gene expression in the tonsils and peripheral blood eosinophils were lower in PFAPA patients compared to controls [11,24]. These results could suggest an ongoing suppression of Th2 responses. We found that levels of CCL5, IL-2 and PDGF were lower in PFAPA-nf than in Pn-nf, and these cytokines were also decreased in children with PFAPA during fever. The relevance of this is, however, not known.

In this study the observed levels of IL-1β in the children with PFAPA were low but elevated in comparison to children with pneumonia. In 2011, Stojanov et al. concluded that PFAPA is a disorder of innate immunity and demonstrated a prompt response to a recombinant IL-1 receptor antagonist in five children with PFAPA [9]. Kolly et al. found increased release of IL-1β from stimulated peripheral blood mononuclear cells and stimulated monocytes in children with PFAPA during febrile episodes [10]. Stojanov et al. and Kolly et al. conclude that IL-1β is likely to have a pivotal role in PFAPA, but they did not find elevated levels of serum IL-1β in the children’s blood samples [9,10]. Our result may to some extent support their theory, but the results are inconclusive.

The haematological pattern, with an increase of total number of white blood cells, neutrophils and monocytes and a decrease of lymphocytes and eosinophils in children during febrile PFAPA episodes, has also been described by others [8,9]. Stojanov et al. found that this pattern differed from children with flares of hereditary periodic fever syndromes, whereas Brown et al. did not include a febrile control group [8,9]. In our study, the lymphocyte counts were lower during febrile episodes in children with PFAPA, but not in children with pneumonia. In children with severe H1N1 pneumonia, serum levels of CXCL10 and IL-6 has been found to be negatively associated with lymphocyte count and disease severity, supporting the role of CXCL10 in recruiting lymphocytes to local tissues [25]. This was not seen in children with community acquired pneumonia. The difference between the two groups in our study may, however, also have been influenced by the different timing of the analyzes.

During severe infections, eosinopenia may be seen as a normal response associated with a Th1 type of inflammation, whereas the absence of eosinopenia has been associated with a Th2 driven response [26,27]. The relative eosinopenia observed by us and others during episodes of PFAPA could therefore be an indication that the inflammation during PFAPA is mainly Th1 driven.

The relative lymphocytopenia corresponds with the decrease in CD4+ and CD8+ subsets of T-lymphocytes, as also described by Stojanov et al. [9]. This is consistent with an activation and redirection of T cells from the bloodstream to local tissue such as lymph nodes and possibly tonsils during febrile episodes in PFAPA. Clinically, this may be seen as the tender cervical adenitis and pharyngitis which are common clinical features of PFAPA [1,2,4-6]. Three studies have reported the results of histological evaluation of tonsils removed from children with PFAPA. They all report signs of chronic inflammation with lymphoid hyperplasia [28-30].

Due to the observed decrease of CD4+ and CD8+ cells in peripheral blood during the febrile episodes of PFAPA, we measured sCD25 in serum. sCD25 is an indicator of T cell activation, and elevated levels have been described in a variety of cancerous and non-cancerous conditions [31-33]. The significant increase of sCD25 observed during the febrile attacks of PFAPA indicates that T cell activation is present, supporting the other results. However, a similar increase during the acute phase of an infection was found in the children with pneumonia.

CD163 is a scavenger receptor involved in the endocytosis of haptoglobin-haemoglobin complexes and is expressed on macrophages and on a fraction of monocytes [34]. Elevated sCD163 has been described in different conditions including hemophagocytic syndrome, sepsis and rheumatoid arthritis [34-36]. To our knowledge sCD163 has not been studied in PFAPA. Levels of sCD163 were not changed during febrile episodes of PFAPA and did not differ between children with PFAPA and children with pneumonia. This may indicate that there is not a pathological over-activation of macrophages in the PFAPA-syndrome.

### Limitations

The diagnostic criteria for PFAPA made by Thomas et al. have a high sensitivity for PFAPA, but do not exclude other hereditary fever syndromes with an overlapping clinical picture. The presence of mutations involved in other genetic fevers has been found in Italian and Israeli children fulfilling the PFAPA criteria [37,38], and a more strict approach to diagnosing PFAPA has been proposed [37]. However, hereditary periodic fever syndromes are uncommon in the Scandinavian population [8], and in the absence of additional characteristics we find it unlikely that we have missed a diagnosis of a hereditary periodic fever syndrome even without performing genetic testing.

We included a control group with pneumonia to study any characteristics of PFAPA different from a common bacterial infection. However, the value of such a control group may be questioned due to the time difference of sampling. In children with PFAPA, the blood sample was taken within the first 24 hours after the onset of a febrile episode. For obvious reasons (they don’t see a doctor this early), this was not possible in the children with pneumonia. It is not unlikely that this time factor influences the measurements and this must be considered when comparing results of the two groups during febrile episodes.

The second sample in children with pneumonia was taken after at least four weeks of complete remission. We therefor consider this sample to be a good negative control, and that the cytokine levels most likely reflect normal base line values in these children.

Clinical studies of relatively uncommon disorders like PFAPA usually have a long running-time in order to collect a reasonable amount of samples. Although seldom debated, this time-factor might influence the result as samples stored over a long period of time might deteriorate [39]. We had to perform one thaw-freeze cycle before sCD25 and sCD163 were analysed and it is possible that this influenced the results of these analyses.

A large number of cytokines were analyzed, which could implicate corrections for multiple comparisons. However, some of the cytokines were randomly included due to their inclusion in the multiplex kit and not by indication. A full Bonferroni correction could therefore be a very conservative approach with the possibility of false negative results (type II error). On the other hand, in the absence of a correction for multiple comparisons there is a risk of false positive result (type I error). Being aware of this, we have interpreted our data with caution and focused on the close resemblance to previous studies. The relatively small sample size should also be considered when interpreting the results of this study.

## Conclusion

We have shown increased levels of cytokines during the febrile attacks of PFAPA, indicating an innate immune response as the initial step. Further, we found a decrease in subsets of T cells, suggesting a subsequent adaptive immune response with activation and redistribution of these cells to local tissue. Levels of CXCL10 were increased in PFAPA also during remission, suggesting a persistent activation of the innate immune system in these children.

## Abbreviations

CRP: C-reactive protein; FGF: Fibroblast growth factor; G-CSF: Granulocyte colony-stimulating factor; GM-CSF: Granulocyte macrophage colony-stimulating factor; Ig: Immunoglobulin; INF: Interferon; IL: Interleukin; PDGF: Platelet-derived growth factor; PFAPA: Periodic fever, aphthous stomatitis, pharyngitis and cervical adenitis syndrome; PFAPA-nf: Non-febrile samples from children with PFAPA; PFAPA-f: Febrile samples from children with PFAPA; Pn-nf: Samples from children with pneumonia after complete resolution; Pn-f: Samples from children with pneumonia taken shortly after admission; sCD25: Soluble IL-2 receptor alpha; TNF: Tumor necrosis factor; VEGF: Vascular endothelial growth factor.

## Competing interests

The authors declare that they have no competing interests.

## Authors’ contributions

All authors were involved in the design of the study. The first draft of the manuscript was written by JF and the manuscript has been continuously reviewed by KØ and EKK. JF is responsible for the collection of data and statistical analyses. EKK was responsible for laboratory analyses at Haukeland University Hospital. All three authors have seen and approved the final version of the manuscript.
